# Closing the Treatment Gap for Lumbar Disc Herniation Patients with Large Annular Defects: A Systematic Review of Techniques and Outcomes in this High-risk Population

**DOI:** 10.7759/cureus.4613

**Published:** 2019-05-07

**Authors:** Joshua Ammerman, William C Watters, Jason A Inzana, Gene Carragee, Michael W Groff

**Affiliations:** 1 Neurosurgery, Sibley Memorial Hospital, Washington, USA; 2 Clinical Orthopedic Surgery, Institute of Academic Medicine, Houston Methodist Hospital, Houston, USA; 3 Orthopaedics, Telos Partners, Denver, USA; 4 Orthopaedic Surgery, Stanford University Medical Center, Stanford, USA; 5 Neurosurgery, Brigham and Women’s Hospital, Boston, USA

**Keywords:** lumbar discectomy, large annular defect, recurrent herniation, reoperation, revision surgery, limited discectomy, subtotal discectomy, annular closure device, lumbar disc herniation, fragmentectomy

## Abstract

Lumbar disc herniation (LDH) is one of the most common spinal pathologies and can be associated with debilitating pain and neurological dysfunction. Discectomy is the primary surgical intervention for LDH and is typically successful. Yet, some patients experience recurrent LDH (RLDH) after discectomy, which is associated with worse clinical outcomes and greater socioeconomic burden. Large defects in the annulus fibrosis are a significant risk factor for RLDH and present a critical treatment challenge. It is essential to identify reliable and cost-effective treatments for this at-risk population. A systematic review of the PubMed and Embase databases was performed according to Preferred Reporting Items for Systematic Reviews and Meta-Analyses (PRISMA) guidelines to identify studies describing the treatment of LDH patients with large annular defects. The incidence of large annular defects, measurement technique, RLDH rate, and reoperation rate were compiled and stratified by surgical technique. The risk of bias was scored for each study and for the identification of RLDH and reoperation. Study heterogeneity and pooled estimates were calculated from the included articles. Fifteen unique studies describing 2,768 subjects were included. The pooled incidence of patients with a large annular defect was 44%. The pooled incidence of RLDH and reoperation following conventional limited discectomy in this population was 10.6% and 6.0%, respectively. A more aggressive technique, subtotal discectomy, tended to have lower rates of RLDH (5.8%) and reoperation (3.8%). However, patients treated with subtotal discectomy reported greater back and leg pain associated with disc degeneration. The quality of evidence was low for subtotal discectomy as an alternative to limited discectomy. Each report had a high risk of bias and treatments were never randomized. A recent randomized controlled trial with 550 subjects examined an annular closure device (ACD) and observed significant reductions in RLDH and reoperation rates (>50% reduction). Based on the available evidence, current discectomy techniques are inadequate for patients with large annular defects, leaving a treatment gap for this high-risk population. Currently, the strongest evidence indicates that augmenting limited discectomy with an ACD can reduce RLDH and revision rates in patients with large annular defects, with a low risk of device complications.

## Introduction and background

Lumbar disc herniation (LDH) is one of the most common spinal pathologies and can be associated with debilitating pain and neurological dysfunction. It is estimated that 500,000 patients undergo surgery for disc herniation annually in the United States, while another one million receive non-operative care [1-2]. Many LDH patients are asymptomatic or minimally symptomatic, but others suffer from intractable pain, numbness, or weakness when the hernia is associated with neural compression.

Management of symptomatic LDH usually progresses through a step-wise non-operative algorithm and may be followed by surgery if symptoms persist for more than six weeks or are associated with neurological deficit or unbearable pain. Even when conservative management is assigned, approximately 40% of patients undergo surgery during the first year of non-operative care [3-4]. Discectomy is the primary surgical intervention for LDH and can be employed through various techniques with the goals of neural decompression and prevention of recurrent herniation. The least aggressive discectomy technique, sequestrectomy or fragmentectomy, removes only the protruding disc without invasion of the intervertebral disc space. The most aggressive technique, subtotal discectomy, removes all protruding or loose material as well as nucleus pulposus from within the annulus and may include endplate curettage. Limited discectomy, which is often regarded as the conventional gold standard technique, is a compromise between sequestrectomy and subtotal discectomy, where the protruding disc and only loose nuclear material from the intervertebral space are removed [5].

In general, discectomy is highly successful and cost-effective for alleviating pain and disability and enabling patients to return to work and their normal daily activities [6-8]. Unfortunately, some patients still experience recurrent LDH (RLDH). Based on a health insurance database analysis of over 7,000 discectomy patients across the United States, the rate of revision discectomy for RLDH within two years of follow-up was 4% [9]. An analysis of nearly 8,000 patients in the Swedish National Spine register (Swespine) also observed a two-year reoperation rate of 4.1% for RLDH [10]. Worse clinical outcomes have been observed for patients who must undergo reoperation (most commonly due to RLDH associated with recurrent symptoms) relative to their non-reoperated counterparts [11-14]. Further, reoperation for RLDH adds substantial direct and indirect costs to the healthcare system [2, 14-15]. The ability to identify high-risk patients and avoid RLDH and reoperation through optimized techniques and innovative technologies is critical to minimize patient morbidity and socioeconomic burden.

A recent meta-analysis of 1,653 lumbar discectomy patients demonstrated that patients with a large annular defect had a significantly increased risk of symptom recurrence (odds ratio (OR) = 2.5, p = 0.004) and reoperation (OR = 2.3, p < 0.001) [16]. Carragee et al. appear to be the first to empirically define and contend that large annular defects are a critical risk factor for RLDH and reoperation [17]. Multiple subsequent studies have further reinforced this concept [18-21]. A large annular defect may be concurrent with the disc herniation or may result from annulotomy during discectomy of contained fragments. Either way, these large defects are readily identifiable intraoperatively and the associated risk of RLDH could be mitigated through appropriate surgical interventions. 

One theory for subtotal discectomy, as compared to limited discectomy or fragmentectomy, is that RLDH is less likely to occur if all of the nuclear material is removed. However, studies have suggested that sacrificing the supporting nuclear material to control RLDH risk could lead to disc space collapse that can translate to spondylosis, abnormal facet loading, and significant back or leg pain [5, 22-26]. The potential tradeoffs of these surgical techniques have left an apparent treatment gap for discectomy patients with large annular defects and a lack of consensus regarding the optimal treatment strategies. In an attempt to resolve this unmet medical need, substantial research and development efforts have pursued the challenge of annular closure or repair in order to reduce the risk of RLDH while preserving the intact nucleus pulposus [27-30]. This study aimed to review the current treatment evidence for lumbar discectomy patients with large annular defects and identify the evidence-based techniques that are most promising for this population.

## Review

Methods

Systematic Literature Review

A systematic review of the PubMed (MEDLINE) and Embase databases was conducted on June 18, 2018 according to the Preferred Reporting Items for Systematic Reviews and Meta-Analyses (PRISMA) guidelines [31] (PRISMA Checklist - Appendix A). The search criteria were chosen to identify articles that describe lumbar discectomy patients with large annular defects and were translated into the relevant syntax for each database (Table 1; Appendix B). 

**Table 1:** Database search terms

Database search terms
Anatomical terms
1. Lumbar
Surgical terms
2. Discectomy
3. Microdiscectomy
4. Sequestrectomy
5. Fragmentectomy
6. Herniotomy
7. Nucleotomy
8. Fragment Excision
9. Annulotomy (Anulotomy)
10. Subtotal
Annular competence terms
11. Annul* (Anul*) – e.g. annular or annulus
12. Defect
13. Competence
14. Tear
15. Size
16. Large
17. Massive
18. Wide or Width
Hernia classification / measurement terms
19. Carragee
20. Fragment-defect
21. Fragment-fissure
22. Fragment-contained
23. Hernia type
24. Fragment type
25. Penfield probe
Term combination strategy
(1) AND (2-10/or) AND (11-25/or)

**Figure 1:**
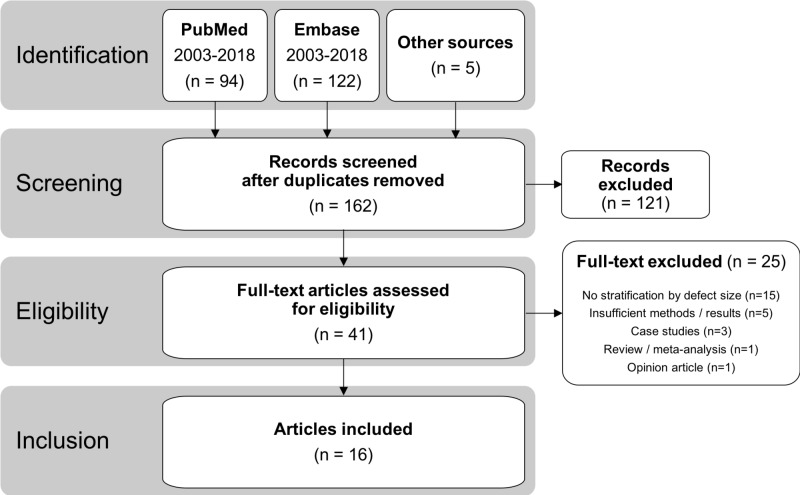
Flowchart of article identification for inclusion in the systematic review according to PRISMA guidelines

The search was limited to articles published in English between the years 2003-2018, based on the first publication by Carragee et al. in 2003 characterizing the high risk of RLDH associated with large defect patients [17]. Bibliographies and review articles were also screened for additional relevant citations. This literature search produced 162 unique articles, which were evaluated by two researchers who screened the titles and abstracts and then applied the eligibility criteria to the remaining full-text articles (Figure 1). Eligibility required that the article was an original report of a clinical study (no pre-clinical research, case reports, systematic reviews, or meta-analyses) that discussed the treatment of lumbar discectomy patients with large annular defects. Data describing stratification based on the defect size measurement or hernia classification criteria along with the resulting RLDH or reoperation rates must have been reported.

Data Compilation and Evaluation

Data on the large annular defect patients from eligible articles were compiled by both researchers to ensure data quality. The methodological and reporting quality of each study was scored using the Methodological Index for Nonrandomized Studies (MINORS) [32]. The risk of bias for the reported RLDH and reoperation rates as well as the classification of a large defect was evaluated for each study by assessing treatment randomization, blinding, and prospectively defined algorithms for determining RLDH or reoperation. The overall risk of bias score was assigned to each study based on the sum of these criteria (“Yes” = 2 points, “Partial or not well described” = 1 point, and “No” or “Not Reported” = 0 points). The risk of bias was rated as “High” if the score was < 33%, “Medium” if the score was ≥ 33% and < 67%, and “Low” if the score was ≥ 67%. 

Data related to the incidence of large annular defects, measurement technique, RLDH rate, and reoperation rate were compiled and sorted according to the surgical technique utilized in each study. The pooled estimate and 95% confidence intervals (CI) were calculated for the incidence of large annular defects as well as the RLDH and reoperation rates. The RLDH and reoperation rates were converted to a proportion per month using mean or standard follow-up times reported by each study. Fragmentectomy and limited discectomy were combined for pooled analyses considering the small sample sizes and similarity of these techniques. Heterogeneity across studies was evaluated using the I^2^ statistic and a random effects model was used for cases of significant heterogeneity (I^2 ^≥ 50%) [33]. Rates of RLDH and reoperation were compared across the different treatment techniques through post-hoc sub-group analyses. All statistical analyses were performed in R software (v3.4.3; Vienna, Austria) at a significance level of p < 0.05.

Results

Study Identification

Sixteen articles describing 15 unique studies met the inclusion criteria for review. Two of the articles described the same clinical trial at two different time points of follow-up [29, 34]. These 15 studies included a total of 2,768 patients treated for a lumbar disc hernia.

Classification and Incidence of Large Annular Defects

The classification of a “large” annular defect was based on intra-operative measurements and/or categorization of hernia type in each of the studies, resulting in a relatively low risk of bias for this metric (Table 2). Three of the studies (19%) used Carragee’s classification system based on intraoperative appearance and characteristics of the hernia and one study (6%) based the classification on chart review of intraoperative notes. Twelve of the 15 studies (80%) used a Penfield probe or dedicated instruments to more objectively measure the defect size intraoperatively (Table 3). The measurement threshold for a large defect was most commonly defined as a 6 mm width (75% of studies) based on the seminal work of Carragee et al., who measured the defects against a Number 1 Penfield probe [17]. Two studies used a threshold of 5 mm and one study used a threshold of 4 mm, but the precision of these measurements is unclear. Ideally, a logistic regression of RLDH risk vs. annular defect size would be performed across a large number of patients, along with interobserver repeatability of the measurements, to identify the potential size threshold for at-risk defects, but no studies have attempted this type of analysis.

**Table 2:** Summary of study reporting quality and potential for bias Abbreviations: Tx, treatment; N/A, not applicable; NR, not reported; RLDH, recurrent lumbar disc herniation; Reop, reoperation Notes: MINORS score consists of two components: a score out of 16 that applies to all studies and a second score out of eight that only applies to comparative studies. These score components are reported separately as (#/16 | #/8) for comparative studies and (#/16 | N/A) for non-comparative studies. The comparative score was only evaluated for treatment comparisons. * These studies reported on various follow-up endpoints and outcomes of the same randomized controlled trial + Independent examiner was used to make the RLDH and reoperation determinations, but blinding was not described ‡ Small defects (≤ 5 mm) were treated with fragmentectomy. Large defects (> 5 mm) were treated with limited discectomy NR = RLDH or reoperation outcomes were not reported N/A = Not applicable due to study type ^&^ We assessed the risk of bias associated with the reported methodology used for determining RLDH or reoperation (2 = low, 1 = medium, 0 = high or not reported) ^ Patients chose between micro-discectomy (subtotal discectomy) and fragmentectomy ^#^ Independent labs, who were blinded to patient outcomes, were used for radiographic analysis. Due to the presence of the device, the radiographic evaluators could not be blinded to the treatment

Study	MINORS Score	Treatment Randomization	Defect Measurement or Hernia Classification	Patient Blinding (Tx)	Follow-up Algorithm^&^		Assessor Blinding (Tx or Defect Size)		Overall Risk of Bias Score (lower score = higher risk)
					RLDH	Reop	RLDH	Reop	
Carragee et al. 2003 [17]	13/16 | N/A	N/A	2	N/A	1	1	1^+^	1^+^	Medium 60% (6/10)
Carragee et al. 2006 [23]	12/16 | 6/8	0	2	N/A	1	1	1^+^	1^+^	Medium 60% (6/10)
Wera et al. 2008 [35]	8/16 | 4/8	0	0	N/A	NR	1	NR	1^+^	High 25% (2/8)
McGirt et al. 2009 [18]	11/16 | N/A	N/A	1	N/A	0	0	1^+^	1^+^	High 30% (3/10)
Kaner et al. 2010 [36]	11/16 | N/A	N/A	1	N/A	0	0	N/A	N/A	High 17% (1/6)
Fakouri et al. 2011 [37]	12/16 | 7/8	0^‡^	2	N/A	0	0	0	0	High 17% (2/12)
Lequin et al. 2012 [38]	12/16 | N/A	N/A	2	N/A	1	1	N/A	N/A	Low 67% (4/6)
Bouma et al. 2013 [39]	13/16 | N/A	N/A	2	N/A	2	1	N/A	N/A	Low 83% (5/6)
Ozer et al. 2013 [40]	9/16 | N/A	N/A	2	N/A	0	NR	N/A	N/A	Medium 50% (2/4)
Kim et al. 2015 [19]	9/16 | N/A	N/A	2	N/A	0	NR	0	NR	Medium 33% (2/6)
Boyaci et al. 2016 [26]	12/16 | 5/8	0^	2	0^	0	0	0	0	High 14% (2/14)
Zhou et al. 2016 [20]	9/16 | N/A	N/A	2	N/A	0	NR	0	NR	Medium 33% (2/6)
Bono et al. 2017 [41]	12/16 | N/A	N/A	1	N/A	0	0	0	0	High 10% (1/10)
Kursumovic et al. 2017 [42]	10/16 | N/A	N/A	2	N/A	1	1	N/A	N/A	Low 67% (4/6)
Klassen et al. 2016 [43]*	Protocol	2	2	1	2	1	1^#^	1^#^	Low 71% (10/14)
Klassen et al. 2017 [34]*	15/16 | 8/8								
Thome et al. 2018 [29]*	15/16 | 8/8								

**Table 3:** Summary of study populations and treatment techniques Abbreviations: Fx, fragmentectomy (also known as sequestrectomy); LD, limited discectomy; SD, subtotal discectomy; PTDS, posterior transpedicular dynamic stabilization; AR, annular repair; ACD, annular closure device; RLDH, recurrent lumbar disc herniation; RCT, randomized controlled trial; ODI, Oswestry disability index; VAS, visual analog scale for pain; N/A, not applicable Notes: ^#^ Incidence could not be estimated in these studies because a large defect was part of the patient inclusion criteria * These studies reported on various follow-up endpoints and outcomes of the same randomized controlled trial

Citation	Study Characteristics			Surgical Technique	Large Defect Characterization		
	Population	Objective	Design		Definition	Technique	Incidence % (n/N)
Carragee et al. 2003 [17]	Sciatica + radicular symptoms; 1-level hernia; 18-65 years	Clinical outcomes based on annular competence	Prospective cohorts	LD	Type II or IV; Width ≥ 6 mm	Penfield Probe	27.2% (49/180)
Carragee et al. 2006 [23]	Sciatica + radicular symptoms; 1-level hernia; 18-65 years; large annular defect	Compare LD vs. SD in large defect patients	Prospective case series (SD) vs. historical control (LD)	SD vs. LD	Type II; Width ≥ 6 mm	Penfield Probe	N/A^# ^(30/30)
Wera et al. 2008 [35]	Sciatica; 1-level lumbar hernia	Compare LD vs. SD	Retrospective case series (SD) vs. historical control (LD)	SD vs. LD	Carragee Type II or IV	Chart Review	56.4% (146/259)
McGirt et al. 2009 [18]	Sciatica + radicular symptoms; 1-level hernia L3-S1; 18-70 yrs; failed ≥6 wks non-op. care	Assess risk factors for RLDH	Prospective cohorts	Fx or LD or SD	Width ≥ 6 mm	Penfield Probe	76.5% (52/68)
Kaner et al. 2010 [36]	Sciatica; 1-level lumbar hernia; 18-60 yrs	Evaluate RLDH rates after LD + PTDS	Prospective case series	LD + PTDS	Carragee Type II or IV	Intra-op Observation	55.0% (22/40)
Fakouri et al. 2011 [37]	1-level hernia L2-S1 + corresponding symptoms; 18-62 yrs	Compare LD (large defect) vs. Fx (small defect)	Retrospective cohorts	LD	≥ 5 mm	Instrument	76.2% (77/101)
Lequin et al. 2012 [38]	Hernia L3-S1; disc height ≥3 mm; failed ≥6 wks non-op. care; ODI and VAS leg ≥40/100; 18-75 yrs	Evaluate safety + efficacy of ACD in large defect patients	Prospective case series	LD + ACD	Width ≥ 6 mm	Dedicated Instruments	N/A^# ^(45/45)
Bouma et al. 2013 [39]	Hernia L3-S1; disc height ≥3 mm; failed ≥6 wks non-op. care; ODI & VAS leg ≥40/100; 18-75 yrs	Evaluate ACD for reducing RLDH in large defect patients	Prospective case series	LD + ACD	Width ≥ 6 mm or Area ≥ 54 mm^2^	Dedicated Instruments	N/A^# ^(65/76)
Ozer et al. 2013 [40]	Sciatica; 1-level lumbar hernia; failed ≥6 wks non-op. care;	Evaluate LD + AR + PTDS in large defect patients	Prospective case series	LD + AR + PTDS	Width ≥ 4 mm	Intra-op Observation	55.2% (54/98)
Kim et al. 2015 [19]	Hernia at L5-S1; 15-78 yrs;	Establish risk factors for RLDH	Retrospective cohorts	LD	Width ≥ 6 mm	Penfield Probe	13.1% (61/467)
Boyaci et al. 2016 [26]	1-level hernia L1-S1; symptoms; 24-65 yrs	Compare SD vs. Fx on RLDH rates	Prospective cohorts	Fx vs. SD	Width ≥ 5 mm	Penfield Probe	37.6% (64/170)
Zhou et al. 2016 [20]	Hernia L3-S1; Mean age 43-46 yrs	Identify risk factors for RLDH	Retrospective cohorts	LD	Width ≥ 6 mm	Penfield Probe	21.0% (86/409)
Bono et al. 2017 [41]	1-level L2-L5 hernia; radicular pain; ≥18 yrs	Evaluate short vs. long restriction of post-op activity	RCT	FX	Carragee Type II or IV	Intra-op Observation	41.0% (41/100)
Kursumovic et al. 2017 [42]	Sciatica; L2-S1 hernia; primary or revision; 18-75 yrs	Evaluate ACD in real-world patients	Prospective case series	LD + ACD	Width 6-10 mm	Dedicated Instruments	N/A^# ^(154/171)
Klassen et al. 2017 [34]*	1-level hernia L2-S1; disc height ≥5 mm; failed ≥6 wks non-op. care; ODI & VAS leg ≥ 40/100; 21-75 years; large defect	Compare LD vs. LD + ACD in large defect patients	RCT	LD vs. LD + ACD	Width 6-10 mm & Height 4-6 mm	Dedicated Instruments	N/A^# ^(554/554)
Thome et al. 2018 [29]*							

**Figure 2:**
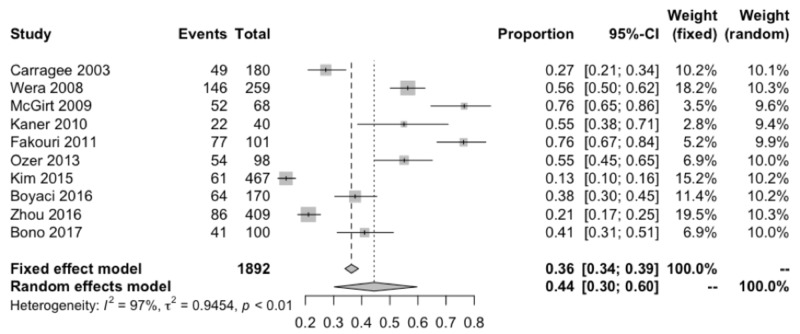
Forest plot of large annular defect incidence Results from the random effects model were used based on the significant heterogeneity (I^2^ = 97%).

Individual studies reported incidence rates of large defects ranging from 13-76%, with a pooled estimate of 44% (95% CI: 30-60%; I^2^=97%; Table 3, Figure 2). This estimate is higher than, but compatible with, a previous meta-analysis on large annular defects that observed a pooled incidence of 30% [16]. None of the included studies prospectively aimed to evaluate large defect incidence. The significant heterogeneity across studies may be attributable to variability in classification or measurement methodology as well as differences in study design that may preselect for different subsets of the discectomy population. The latter would constitute a selection bias. For example, Wera et al. retrospectively reviewed chart data from 1100 discectomy cases, but ultimately could only include 259 (24%) cases in the analysis [35]. Additionally, Boyaci et al. cited difficulties in identifying the type of hernias and defect sizes as limitations to their study [26]. Incidence data from studies of an annular closure device (ACD) were excluded from this analysis because a large defect was generally one of the inclusion criteria. A prospective multi-center study with a focus on the incidence of large annular defects would be important for a reliable estimate among the general discectomy population.

Outcome Bias

There was a risk of bias for reherniation and reoperation outcomes in most of the studies, which was largely attributable to a lack of systematic methodology, blinding, or sufficient reporting (Table 2). A clear, prospective algorithm for defining RLDH is ideal since significant variability can exist. For example, index-level RLDH may be reported for both contralateral and ipsilateral events or just ipsilateral events. The extent to which patient symptoms, radiological findings, or intra-operative confirmation are considered in the RLDH definition is also important. Only two studies (three citations) prospectively defined and reported such details for RLDH and one published a prospective protocol [29, 34, 39, 43]. The choice to reoperate is also susceptible to bias, which may be unavoidable due to patient preferences and ethical considerations that make an objective decision algorithm challenging. Blinding of patients and investigators is possible in prospective studies, but complete patient blinding was not utilized in either of the two prospective studies reviewed herein. Boyaci et al. allowed patients to choose between subtotal discectomy and fragmentectomy [26]. The randomized controlled trial (RCT) comparing ACD and limited discectomy could only blind a subset of patients due to regional allowances [29]. In that RCT, radiological assessment could not be fully blinded due to the presence of the ACD, but independent radiologists were blinded to the patient outcomes.

Comparative Analysis of Treatment Techniques and Outcomes

Among the 15 unique studies, 19 unique treatment cohorts were described. Five of those patient cohorts were treated with limited discectomy, four were treated with subtotal discectomy, two with fragmentectomy, and one study reported on patients treated with any of these three discectomy techniques without delineation (Table 4). An additional seven studies reported on patients treated with limited discectomy augmented by either dynamic transpedicular screw stabilization (two studies) or an ACD (five studies). At least three studies per treatment type were necessary to calculate pooled estimates, so fragmentectomy was combined with limited discectomy considering the small number of studies and the similarity of these techniques. The two studies on dynamic transpedicular screw stabilization could not be included in the pooled estimates and are described separately.

**Table 4:** Summary of RLDH and reoperation outcomes by surgical technique Abbreviations: RLDH, recurrent lumbar disc herniation; SD, standard deviation Notes: ^#^ RLDH rates are reported for large annular defect patients only * These four studies reported on various follow-up endpoints and outcomes of the same randomized controlled trial

Surgical Technique	Citation	Follow-up Period	RLDH Rate^#^ % (n/N)	Reoperation Rate % (n/N)
Fragmentectomy	Boyaci et al. 2016 [29]	Mean ± SD: 34 ± 5 months	0% (0/27)	0% (0/27)
	Bono et al. 2017 [44]	12 months	9.8% (4/41)	2.4% (1/41)
Limited Discectomy	Carragee et al. 2003 [20]	Min: 2 years; Median: 6 years	22.4% (11/49)	16.3% (8/49)
	Kim et al. 2015 [22]	Mean ± SD: 51 ± 23 months	18.0% (11/61)	Not Reported
	Zhou et al. 2016 [23]	>12 months	15.1% (13/86)	Not Reported
	Klassen et al. 2017 [37]*	90 days	6.8% (19/278)	4.0% (11/278)
	Thome et al. 2018 [32]*	24 months	25.3% (65/257)	13.3% (37/278)
Subtotal Discectomy	Carragee et al. 2006 [26]	24 months	6.7% (2/30)	6.7% (2/30)
	Wera et al. 2008 [38]	Mean: 98 months (Range: 2-305 months)	Not Reported	3.3% (2/60)
	Fakouri et al. 2011 [40]	Mean ± SD: 32 ± 6 months	5.6% (4/72)	5.6% (4/72)
	Boyaci et al. 2016 [29]	Mean ± SD: 34 ± 5 months	10.8% (4/37)	10.8% (4/37)
Variable techniques (Fragmentectomy to Subtotal Discectomy)	McGirt et al. 2009 [21]	Mean ± SD: 25 ± 12 months	11.5% (6/52)	11.5% (6/52)
Limited Discectomy + Posterior Stabilization	Kaner et al. 2010 [39]	24 months	0% (0/22)	0% (0/22)
Limited Discectomy + Annular Closure Device	Lequin et al. 2012 [41]	12 months	2.4% (1/41)	2.4% (1/41)
	Bouma et al. 2013 [42]	Mean: 18.7 months; Median: 24 months	1.3% (1/75)	1.3% (1/75)
	Kursumovic et al. 2017 [45]	Mean: 15 months (Range: 1-72 months)	3.5% (6/171)	2.3% (4/171)
	Klassen et al. 2017 [37]*	90 days	2.2% (6/272)	0.7% (2/272)
	Thome et al. 2018 [32]*	24 months	12.4% (31/250)	5.1% (14/272)
Limited Discectomy + Annular Repair + Posterior Stabilization	Ozer et al. 2013 [43]	Not Reported	5.5% (3/54)	Not Reported

Only six of the 15 studies presented a comparative analysis of treatment techniques. Wera et al. and Carragee et al. described retrospective and prospective cohorts treated with subtotal discectomy that were compared to the historical limited discectomy cohort described by Carragee et al. in 2003 [17, 23, 35]. Both of these studies reported significantly lower rates of RLDH and reoperation with subtotal discectomy versus limited discectomy; however, Carragee et al. reported significantly worse pain and disability scores at one-year follow-up, less patient satisfaction, and slower return to work in the subtotal discectomy population. Fakouri et al. retrospectively observed comparable outcomes between limited discectomy and fragmentectomy, but limited discectomy was only performed on large defect patients and fragmentectomy on small defect patients [37]. Boyaci et al. compared subtotal discectomy and fragmentectomy in non-randomized prospective cohorts and observed similar reoperation rates, but worse disability scores in the subtotal discectomy group [26]. Instead of randomization in that series, the patients were informed of the two surgery options and were allowed to choose the surgical technique. Finally, two studies reported on the 90-day and two-year outcomes from a RCT of limited discectomy alone (Control) versus limited discectomy augmented with an ACD. These studies observed that the ACD significantly reduced RLDH and reoperation rates by 52% and 62%, respectively [29, 34].

**Figure 3:**
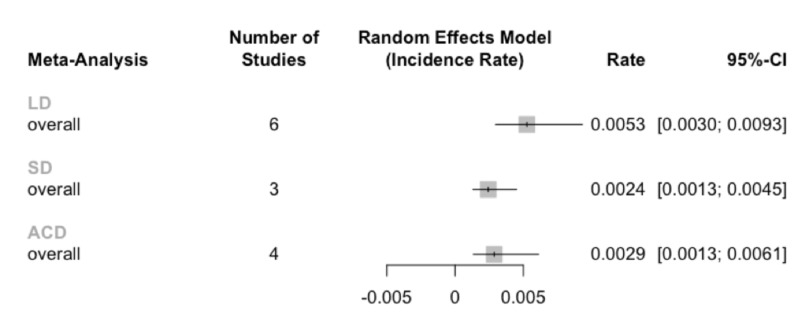
Unpaired meta-analysis of the RLDH rates Rates are reported per month, so multiplication by 24 months yields the pooled two-year RLDH rates. Abbreviations: limited discectomy (LD); subtotal discectomy (SD); annular closure device (ACD).

Due to the low number and characteristics of the available comparative studies, a paired meta-analysis of the surgical techniques could not be completed. The pooled two-year RLDH rate for limited discectomy / fragmentectomy was 10.6% (95% CI: 6.0-18.6%; I^2^=81%), subtotal discectomy was 5.8% (95% CI: 3.1-10.8%; I^2^=0%), and ACD was 7.0% (95% CI: 3.1-14.6%; I^2^=57%). Unpaired meta-analysis with subgroup comparisons (which breaks any pairing or randomization) was insufficiently powered to detect any significant differences in RLDH rates between the three treatment types (p=0.17; Figure 3).

**Figure 4:**
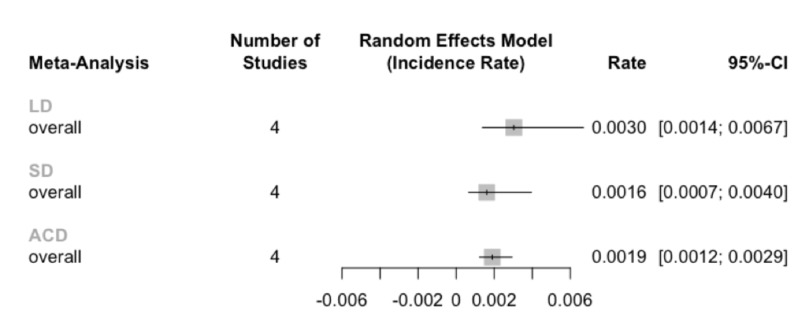
Unpaired meta-analysis of the reoperation rates Rates are reported per month, so multiplication by 24 months yields the two-year pooled reoperation rates. Abbreviations: limited discectomy (LD); subtotal discectomy (SD); annular closure device (ACD).

The pooled two-year reoperation rate for limited discectomy was 6.0% (95% CI: 2.8-13.4%; I^2^=64%), subtotal discectomy was 3.8% (95% CI: 1.7-9.6%; I^2^=58%), and ACD was 4.6% (95% CI: 2.9-7.0%; I^2^=0%). Unpaired meta-analysis with subgroup comparisons was insufficiently powered to detect any significant differences in reoperation rates between the three treatment types (p=0.57; Figure 4).

Discussion

Performing limited discectomy or fragmentectomy on lumbar herniation patients with large annular defects resulted in two-year RLDH and reoperation rates of 10.6% and 6.0%, respectively, across the reviewed studies. Alternative treatment strategies identified in this literature review included subtotal discectomy or augmenting limited discectomy with dynamic posterior transpedicular screw stabilization or an ACD. The two studies that employed dynamic transpedicular posterior stabilization in addition to limited discectomy observed RLDH rates of 4.3% and 0% [36, 40]. In addition to the posterior instrumentation, Ozer et al. attempted annular repair through bipolar cauterization, which may have also influenced the RLDH rate [40]. Dynamic pedicle screw systems are much more invasive than discectomy alone or utilization of an ACD and can result in unintended facet arthrodesis [44-45]. Pedicle screw stabilization and arthrodesis are more appropriate for cases of segmental instability and are unlikely to constitute an ideal default approach for supplementing discectomy in large defect patients [46].

McGirt et al. measured the volume of disc material removed during procedures ranging from fragmentectomy to subtotal discectomy and observed that patients with less disc removed and larger annular defects were at significantly greater risk for RLDH [18]. This finding is consistent with the philosophy of subtotal discectomy, which aims to reduce the risk of RLDH by leaving behind less material to potentially reherniate in the future. Multiple studies have examined subtotal versus limited discectomy in the more general population and observed a lower incidence of RLDH with subtotal discectomy [22-24, 35]. In contrast to limited discectomy, subtotal discectomy has also been associated with significantly worse leg and back pain [22-24]. The liberal removal of the nucleus with aggressive discectomy techniques could accelerate disc space collapse, resulting in spondylosis, abnormal facet loading, and recurrent pain [5, 25]. This trade-off suggests that the two techniques may have different advantages and disadvantages, but both may leave large annular defect patients at risk for future pain and disability. 

In an effort to avoid the trade-offs of limited vs. subtotal discectomy, four annular closure or repair devices have been introduced commercially to help avoid RLDH following limited discectomy. These devices include: a mesh implant possessing two annular suture assemblies (Inclose^TM^ Surgical Mesh System; Anulex Technologies, Inc., Minnetonka, MN); an annular suture kit (AnchorKnot® Tissue Approximation Kit; Anchor Orthopedics, Mississauga, Ontario, Canada); a polyetheretherketone (PEEK) implant that is secured to the apophyseal ring at the posterior vertebral edge (The DART System; Magellan Spine Technologies, Inc., Irvine, CA); and a polymeric component secured to a titanium base, which anchors into the vertebral body and occludes the annular defect (Barricaid®; Intrinsic Therapeutics, Inc., Woburn, MA). The Barricaid® ACD was the subject of a large (550 subjects) multicenter RCT that achieved reductions of 52% in symptomatic RLDH rates and 62% in revisions for RLDH compared to limited discectomy alone [29, 34]. Thus far, this ACD has also proven to be safe, with a low rate of device-related revisions (4/272 patients; 1.5%) [14, 29]. Radiographic vertebral endplate changes were observed at a higher rate in the ACD group than the Control group but were not associated with clinical outcomes in the ACD group [47]. Low RLDH, reoperation, and complication rates with this ACD have also been supported by registry analyses of real-world patients and other case series [38-39, 42, 48]. A formal cost-utility analysis of the ACD versus discectomy alone determined the incremental cost-effectiveness ratio (ICER) to be only $6,030 per quality-adjust life year (QALY) for direct medical costs. If indirect costs, such as productivity loss, were also considered, the ICER for ACD compared to discectomy alone was actually negative, which indicates that greater quality of life was achieved at a lower cost-a situation referred to as “economic dominance” [49]. As a comparison, an economic analysis of the Spine Patient Outcomes Research Trial (SPORT) observed an ICER of $69,403 per QALY for all costs of decompression or discectomy versus non-operative treatment for lumbar disc herniation [7].

The current study’s attempts at meta-analysis and subgroup comparisons between limited discectomy, subtotal discectomy, and ACD were underpowered and pairing/randomization needed to be broken. Miller et al. conducted a meta-analysis on large vs. small annular defects and also found that subgroup analyses of different treatment techniques were too underpowered to yield conclusive results [16]. The large multicenter RCT comparing limited discectomy alone versus augmentation with an ACD was the only high-quality comparative evidence available for lumbar herniation patients with large annular defects. Cohort meta-analysis in the current study suggested that subtotal discectomy may lower the rates of RLDH relative to limited discectomy, but the quality of evidence for this conclusion is very low for large defect patients as well as the more general discectomy population [50]. This sparsity of quality evidence, combined with the potential side effects of subtotal discectomy, suggests that subtotal discectomy should not be recommended as an alternative to limited discectomy without further data to inform the benefit-risk profile.

## Conclusions

An unmet medical need is a condition that is not addressed adequately by available therapy and includes an immediate need for a defined population. LDH patients presenting with a large annular defect are not adequately treated by conventional discectomy techniques and constitute a readily identifiable at-risk population based on intraoperative annular defect measurement. In this review of the literature, the current standard treatment (limited discectomy) has not adequately addressed the high risk of symptomatic RLDH within this population. Subtotal discectomy tends to trade RLDH risk for disc degeneration and new sources of pain. The strongest evidence to date for treatment of this high-risk population indicates that augmenting limited discectomy with an ACD can reduce RLDH and revision rates by more than 50% with a low risk of device complications and a promising cost-effectiveness profile.

## Appendices

Appendix A: PRISMA checklist

**Table 5:** PRISMA 2009 Checklist

Section/topic	#	Checklist item	Reported on page #
TITLE			
Title	1	Identify the report as a systematic review, meta-analysis, or both.	1
ABSTRACT			
Structured summary	2	Provide a structured summary including, as applicable: background; objectives; data sources; study eligibility criteria, participants, and interventions; study appraisal and synthesis methods; results; limitations; conclusions and implications of key findings; systematic review registration number.	2
INTRODUCTION			
Rationale	3	Describe the rationale for the review in the context of what is already known.	3-4
Objectives	4	Provide an explicit statement of questions being addressed with reference to participants, interventions, comparisons, outcomes, and study design (PICOS).	4
METHODS			
Protocol and registration	5	Indicate if a review protocol exists, if and where it can be accessed (e.g., Web address), and, if available, provide registration information including registration number.	N/A
Eligibility criteria	6	Specify study characteristics (e.g., PICOS, length of follow-up) and report characteristics (e.g., years considered, language, publication status) used as criteria for eligibility, giving rationales.	4-5
Information sources	7	Describe all information sources (e.g., databases with dates of coverage, contact with study authors to identify additional studies) in the search and date last searched.	4-5
Search	8	Present full electronic search strategy for at least one database, including any limits used, such that it could be repeated.	Table 1, Appendix
Study selection	9	State the process for selecting studies (i.e., screening, eligibility, included in systematic review, and, if applicable, included in the meta-analysis).	5
Data collection process	10	Describe method of data extraction from reports (e.g., piloted forms, independently, in duplicate) and any processes for obtaining and confirming data from investigators.	5
Data items	11	List and define all variables for which data were sought (e.g., PICOS, funding sources) and any assumptions and simplifications made.	5, Tables 2-3
Risk of bias in individual studies	12	Describe methods used for assessing risk of bias of individual studies (including specification of whether this was done at the study or outcome level), and how this information is to be used in any data synthesis.	5
Summary measures	13	State the principal summary measures (e.g., risk ratio, difference in means).	5
Synthesis of results	14	Describe the methods of handling data and combining results of studies, if done, including measures of consistency (e.g., I^2^) for each meta-analysis.	5
Risk of bias across studies	15	Specify any assessment of risk of bias that may affect the cumulative evidence (e.g., publication bias, selective reporting within studies).	5
Additional analyses	16	Describe methods of additional analyses (e.g., sensitivity or subgroup analyses, meta-regression), if done, indicating which were pre-specified.	5-6
RESULTS			
Study selection	17	Give numbers of studies screened, assessed for eligibility, and included in the review, with reasons for exclusions at each stage, ideally with a flow diagram.	5, Figure 1
Study characteristics	18	For each study, present characteristics for which data were extracted (e.g., study size, PICOS, follow-up period) and provide the citations.	Tables 3-4
Risk of bias within studies	19	Present data on risk of bias of each study and, if available, any outcome level assessment (see item 12).	Table 2
Results of individual studies	20	For all outcomes considered (benefits or harms), present, for each study: (a) simple summary data for each intervention group (b) effect estimates and confidence intervals, ideally with a forest plot.	Table 4
Synthesis of results	21	Present results of each meta-analysis done, including confidence intervals and measures of consistency.	11-15
Risk of bias across studies	22	Present results of any assessment of risk of bias across studies (see Item 15).	11-12
Additional analysis	23	Give results of additional analyses, if done (e.g., sensitivity or subgroup analyses, meta-regression [see Item 16]).	13-15
DISCUSSION			
Summary of evidence	24	Summarize the main findings including the strength of evidence for each main outcome; consider their relevance to key groups (e.g., healthcare providers, users, and policy makers).	15-17
Limitations	25	Discuss limitations at study and outcome level (e.g., risk of bias), and at review-level (e.g., incomplete retrieval of identified research, reporting bias).	17
Conclusions	26	Provide a general interpretation of the results in the context of other evidence, and implications for future research.	17
FUNDING			
Funding	27	Describe sources of funding for the systematic review and other support (e.g., supply of data); role of funders for the systematic review.	19

Appendix B: Database search criteria

Embase

('lumbar spine'/exp OR 'lumbar' OR 'lumbar spine') AND ('diskectomy' OR 'discectomy'/exp OR discectom* OR microdiscectom* OR microdiskectom* OR sequestrectom* OR fragmentectom* OR herniotom* OR nucleotom* OR annulotom* OR anulotom* OR 'fragment excision' OR subtotal) AND (competence OR defect OR 'tear'/exp OR tear OR anul* OR annul*) AND ('size'/exp OR size OR large OR massive OR wide OR 'width'/exp OR width OR carragee OR 'fragment defect' OR 'fragment fissure' OR 'fragment contained' OR 'fragment-defect' OR 'fragment-fissure' OR 'fragment-contained' OR 'penfield probe' OR 'herniation type' OR 'hernia type' OR 'fragment type')

Pubmed/MEDLINE

(lumbar OR lumbar spine) AND (diskectomy OR discectom* OR microdiscectom* OR microdiskectom* OR sequestrectom* OR fragmentectom* OR herniotom* OR nucleotom* OR annulotom* OR anulotom* OR fragment excision OR subtotal) AND (competence OR defect OR tear OR anul* OR annul*) AND (size OR large OR massive OR wide OR width OR carragee OR fragment defect OR fragment fissure OR fragment contained OR fragment defect OR fragment fissure OR fragment contained OR penfield probe OR herniation type OR hernia type OR fragment type)

Filters: January 1, 2003-June 18, 2008

## References

[1] Gray DT, Deyo RA, Kreuter W, Mirza SK, Heagerty PJ, Comstock BA, Chan L (2006). Population-based trends in volumes and rates of ambulatory lumbar spine surgery. Spine (Phila Pa 1976).

[2] Sherman J, Cauthen J, Schoenberg D, Burns M, Reaven NL, Griffith SL (2010). Economic impact of improving outcomes of lumbar discectomy. Spine J.

[3] Peul WC, van Houwelingen HC, van den Hout WB (2007). Surgery versus prolonged conservative treatment for sciatica. N Engl J Med.

[4] Weinstein JN, Tosteson TD, Lurie JD (2006). Surgical vs nonoperative treatment for lumbar disk herniation: the Spine Patient Outcomes Research Trial (SPORT): a randomized trial. JAMA.

[5] Fakouri B, Shetty NR, White TC (2015). Is sequestrectomy a viable alternative to microdiscectomy? A systematic review of the literature. Clin Orthop Relat Res.

[6] Lewis R, Williams N, Matar HE (2011). The clinical effectiveness and cost-effectiveness of management strategies for sciatica: systematic review and economic model. Health Technol Assess.

[7] Tosteson AN, Skinner JS, Tosteson TD (2008). The cost effectiveness of surgical versus nonoperative treatment for lumbar disc herniation over two years: evidence from the Spine Patient Outcomes Research Trial (SPORT). Spine (Phila Pa 1976).

[8] Weinstein JN, Lurie JD, Tosteson TD (2008). Surgical versus nonoperative treatment for lumbar disc herniation: four-year results for the Spine Patient Outcomes Research Trial (SPORT). Spine (Phila Pa 1976).

[9] Virk SS, Diwan A, Phillips FM, Sandhu H, Khan SN (2017). What is the rate of revision discectomies after primary discectomy on a national scale?. Clin Orthop Relat Res.

[10] Elkan P, Lagerback T, Moller H, Gerdhem P (2018). Response rate does not affect patient-reported outcome after lumbar discectomy. Eur Spine J.

[11] Leven D, Passias PG, Errico TJ (2015). Risk factors for reoperation in patients treated surgically for intervertebral disc herniation: a subanalysis of eight-year SPORT data. J Bone Joint Surg Am.

[12] Abdu RW, Abdu WA, Pearson AM, Zhao W, Lurie JD, Weinstein JN (2017). Reoperation for recurrent intervertebral disc herniation in the Spine Patient Outcomes Research Trial: analysis of rate, risk factors and outcome. Spine (Phila Pa 1976).

[13] Fritzell P, Knutsson B, Sanden B, Stromqvist B, Hagg O (2015). Recurrent versus primary lumbar disc herniation surgery: patient-reported outcomes in the Swedish Spine Register Swespine. Clin Orthop Relat Res.

[14] Klassen PD, Hsu WK, Martens F, Inzana JA, van den Brink WA, Groff MW, Thomé C (2018). Post-lumbar discectomy reoperations that are associated with poor clinical and socioeconomic outcomes can be reduced through use of a novel annular closure device: results from a 2-year randomized controlled trial. Clinicoecon Outcomes Res.

[15] O'Donnell JA, Anderson JT, Haas AR, Percy R, Woods ST, Ahn UM, Ahn NU (2017). Treatment of recurrent lumbar disc herniation with or without fusion in workers' compensation subjects. Spine (Phila Pa 1976).

[16] Miller LE, McGirt MJ, Garfin SR, Bono CM (2018). Association of annular defect width after lumbar discectomy with risk of symptom recurrence and reoperation: systematic review and meta-analysis of comparative studies. Spine (Phila Pa 1976).

[17] Carragee EJ, Han MY, Suen PW, Kim D (2003). Clinical outcomes after lumbar discectomy for sciatica: the effects of fragment type and anular competence. J Bone Joint Surg Am.

[18] McGirt MJ, Eustacchio S, Varga P (2009). A prospective cohort study of close interval computed tomography and magnetic resonance imaging after primary lumbar discectomy: factors associated with recurrent disc herniation and disc height loss. Spine (Phila Pa 1976).

[19] Kim KT, Lee DH, Cho DC, Sung JK, Kim YB (2015). Preoperative risk factors for recurrent lumbar disk herniation in L5-S1. J Spinal Disord Tech.

[20] Zhou BW, Wang K, Hong X (2016). Adjacent level disc degeneration: a prognostic factor for recurrent lumbar disc herniation after transforaminal endoscopic lumbar discectomy in 409 cases. Int J Clin Exp Med.

[21] Huang W, Han Z, Liu J, Yu L, Yu X (2016). Risk factors for recurrent lumbar disc herniation: a systematic review and meta-analysis. Medicine (Baltimore).

[22] McGirt MJ, Ambrossi GL, Datoo G (2009). Recurrent disc herniation and long-term back pain after primary lumbar discectomy: review of outcomes reported for limited versus aggressive disc removal. Neurosurgery.

[23] Carragee EJ, Spinnickie AO, Alamin TF, Paragioudakis S (2006). A prospective controlled study of limited versus subtotal posterior discectomy: short-term outcomes in patients with herniated lumbar intervertebral discs and large posterior anular defect. Spine (Phila Pa 1976).

[24] Watters WC 3rd, McGirt MJ (2009). An evidence-based review of the literature on the consequences of conservative versus aggressive discectomy for the treatment of primary disc herniation with radiculopathy. Spine J.

[25] Mochida J, Nishimura K, Nomura T, Toh E, Chiba M (1996). The importance of preserving disc structure in surgical approaches to lumbar disc herniation. Spine (Phila Pa 1976).

[26] Boyaci S, Aksoy K (2016). Long-term clinical outcome of the lumbar microdiscectomy and fragmentectomy: A prospective study. Neurosurg Q.

[27] Guterl CC, See EY, Blanquer SB (2019). Challenges and strategies in the repair of ruptured annulus fibrosus. Eur Cell Mater.

[28] Bailey A, Araghi A, Blumenthal S, Huffmon GV, Anular Repair Clinical Study Group (2013). Prospective, multicenter, randomized, controlled study of anular repair in lumbar discectomy: two-year follow-up. Spine (Phila Pa 1976).

[29] Thomé C, Klassen PD, Bouma GJ (2018). Annular closure in lumbar microdiscectomy for prevention of reherniation: a randomized clinical trial. Spine J.

[30] Qi L, Li M, Si H, Wang L, Jiang Y, Zhang S, Li L (2016). The clinical application of "jetting suture" technique in annular repair under microendoscopic discectomy: A prospective single-cohort observational study. Medicine (Baltimore).

[31] Moher D, Liberati A, Tetzlaff J, Altman DG, PRISMA Group (2019). Preferred reporting items for systematic reviews and meta-analyses: the PRISMA statement. PLoS Med.

[32] Slim K, Nini E, Forestier D, Kwiatkowski F, Panis Y, Chipponi J (2003). Methodological index for nonrandomized studies (MINORS)- development and validation of a new instrument. ANZ J Surg.

[33] Higgins JP, Thompson SG, Deeks JJ, Altman DG (2003). Measuring inconsistency in meta-analyses. BMJ.

[34] Klassen PD, Bernstein DT, Kohler HP, Arts MP, Weiner B, Miller LE, Thomé C (2019). Bone-anchored annular closure following lumbar discectomy reduces risk of complications and reoperations within 90 days of discharge. J Pain Res.

[35] Wera GD, Dean CL, Ahn UM, Marcus RE, Cassinelli EH, Bohlman HH, Ahn NU (2008). Reherniation and failure after lumbar discectomy: a comparison of fragment excision alone versus subtotal discectomy. J Spinal Disord Tech.

[36] Kaner T, Sasani M, Oktenoglu T, Cosar M, Ozer AF (2019). Clinical outcomes after posterior dynamic transpedicular stabilization with limited lumbar discectomy: Carragee classification system for lumbar disc herniations. SAS J.

[37] Fakouri B, Patel V, Bayley E, Srinivas S (2011). Lumbar microdiscectomy versus sequesterectomy/free fragmentectomy: a long-term (2 y) retrospective study of the clinical outcome. J Spinal Disord Tech.

[38] Lequin MB, Barth M, Thomé C, Bouma GJ (2012). Primary limited lumbar discectomy with an annulus closure device: one-year clinical and radiographic results from a prospective, multi-center study. Korean J Spine.

[39] Bouma GJ, Barth M, Ledic D, Vilendecic M (2013). The high-risk discectomy patient: prevention of reherniation in patients with large anular defects using an anular closure device. Eur Spine J.

[40] Ozer AF, Keskin F, Oktenoglu T, Suzer T, Ataker Y, Gomleksiz C, Sasani M (2013). A novel approach to the surgical treatment of lumbar disc herniations: indications of simple discectomy and posterior transpedicular dynamic stabilization based on carragee classification. Adv Orthop.

[41] Bono CM, Leonard DA, Cha TD, Schwab JH, Wood KB, Harris MB, Schoenfeld AJ (2017). The effect of short (2-weeks) versus long (6-weeks) post-operative restrictions following lumbar discectomy: a prospective randomized control trial. Eur Spine J.

[42] Kursumovic A, Rath S (2017). Performance of an annular closure device in a 'real-world', heterogeneous, at-risk, lumbar discectomy population. Cureus.

[43] Klassen PD, Hes R, Bouma GJ (2016). A multicenter, prospective, randomized study protocol to demonstrate the superiority of a bone-anchored prosthesis for anular closure used in conjunction with limited discectomy to limited discectomy alone for primary lumbar disc herniation. Int J Clin Trials.

[44] Fay LY, Chang PY, Wu JC (2016). Dynesys dynamic stabilization-related facet arthrodesis. Neurosurg Focus.

[45] Yeh MY, Kuo CH, Wu JC (2018). Changes of facet joints after dynamic stabilization: continuous degeneration or slow fusion?. World Neurosurg.

[46] Thomé C, Borm W, Meyer F (2008). Degenerative lumbar spinal stenosis: current strategies in diagnosis and treatment. Dtsch Arztebl Int..

[47] Kursumovic A, Kienzler JC, Bouma GJ (2018). Morphology and clinical relevance of vertebral endplate changes following limited lumbar discectomy with or without bone-anchored annular closure. Spine (Phila Pa 1976).

[48] Kursumovic A, Rath SA (2018). Effectiveness of an annular closure device in a "real-world" population: stratification of registry data using screening criteria from a randomized controlled trial. Med Devices (Auckl).

[49] Ament J, Yang Z, Thaci B, Kulubya E, Hsu W, Bouma G, Kim KD (2018). Cost-effectiveness of a bone-anchored annular closure device versus conventional lumbar discectomy in treating lumbar disc herniations. Spine (Phila Pa 1976).

[50] Azarhomayoun A, Chou R, Shirdel S, Lakeh MM, Vaccaro AR, Rahimi-Movaghar V (2015). Sequestrectomy versus conventional microdiscectomy for the treatment of a lumbar disc herniation: a systematic review. Medicine (Baltimore).

